# Jaceosidin Attenuates Sepsis-Induced Myocardial Dysfunction by Promoting SIRT2-Mediated Inhibition of Histone H3K18 Lactylation

**DOI:** 10.3390/ph19010097

**Published:** 2026-01-04

**Authors:** Huiming Yu, Minfu Liu, Shuwan Hou, Jiaqin Wu, Qianqian Du, Fan Feng, Sixiang Wang, Chunli Wang, Kang Xu

**Affiliations:** 1 Hubei Provincial Engineering Technology Research Center for Chinese Medicine Processing, School of Pharmacy, Hubei University of Chinese Medicine, Wuhan 430065, China; huimingyu1213@stmail.hbucm.edu.cn (H.Y.); qianqiandu610@stmail.hbucm.edu.cn (Q.D.); ffan2024@hbucm.edu.cn (F.F.); sixiangwang2025@163.com (S.W.); 2 School of Laboratory Medicine, Hubei University of Chinese Medicine, Wuhan 430065, China; 2432800029@stmail.hbucm.edu.cn (M.L.); shuwanhou222@stmail.hbucm.edu.cn (S.H.); jiaqin_wu@cqu.edu.cn (J.W.); 3 Hubei Shizhen Laboratory, Wuhan 430065, China

**Keywords:** Jaceosidin, sepsis-induced myocardial dysfunction, H3K18la, SIRT2, lactylation

## Abstract

**Background:** Sepsis-induced myocardial dysfunction (SIMD) is a life-threatening complication with limited therapeutic options. Jaceosidin (JAC), a natural flavonoid from Folium Artemisiae Argyi, shows potential in cardiovascular diseases, but its role and mechanism in SIMD remain unclear. This study aims to investigate the protective effects of JAC against SIMD and explore the underlying molecular mechanisms. **Methods:** In vitro, AC16 human cardiomyocytes were stimulated with TNF-α and treated with JAC. Cell viability and apoptosis were assessed using CCK−8 and flow cytometry, respectively. Transcriptomic and metabolomic analyses were performed to identify altered pathways. Molecular docking evaluated JAC’s interaction with SIRT2. The SIRT2 inhibitor AGK2 was used to validate its role. Chromatin immunoprecipitation quantitative PCR (ChIP-qPCR) determined H3K18la enrichment on target gene promoters. In vivo, a murine SIMD model was established via LPS injection, and cardiac function was evaluated by echocardiography. Serum markers (cTnT, CK−MB) and myocardial lactylation levels were measured. **Results:** JAC significantly attenuated TNF-α−induced injury in AC16 cells by enhancing viability and reducing apoptosis. Multi-omics analyses revealed JAC suppressed glycolysis and lactate production. JAC specifically inhibited histone H3K18 lactylation (H3K18la), and molecular docking indicated strong binding affinity with SIRT2. AGK2 treatment reversed JAC-mediated suppression of H3K18la. ChIP-qPCR confirmed *H3K18la* directly regulates *IL-6*, *BAX*, and *BCL-2* expression. In vivo, JAC improved cardiac function (LVEF, LVFS, LVDd, LVDs), reduced serum cTnT and CK−MB levels, and decreased myocardial H3K18la in LPS−treated mice. **Conclusions:** JAC alleviates SIMD by activating SIRT2, which inhibits H3K18la, thereby modulating inflammatory and apoptotic pathways. This study identifies JAC as a novel metabolic-epigenetic therapeutic agent for SIMD.

## 1. Introduction

Sepsis is a life-threatening organ dysfunction syndrome caused by a dysregulated host response to infection, with the heart being one of the key target organs highly vulnerable to such dysregulation and injury [1]. Sepsis-induced myocardial dysfunction (SIMD) is a frequent and severe complication in septic patients, associated with increased mortality [2]. Substantial evidence indicates that approximately 50% of septic patients exhibit signs of myocardial dysfunction [3]. The pathogenesis of SIMD is highly complex, entailing multifactorial synergism and collaborative effects among dysregulated inflammatory responses, mitochondrial dysfunction, calcium dyshomeostasis, apoptosis/pyroptosis, and metabolic derangements [4,5]. Notably, this multifactorial pathogenesis aligns with the traditional medicine concept of “toxic heat invading the heart collaterals”, wherein systemic infection triggers inflammatory storm and oxidative stress, leading to qi stagnation, blood stasis, and phlegm turbidity obstructing the heart vessels. Pathogen-associated molecular patterns (PAMPs), such as lipopolysaccharide (LPS)—an exotoxin from Gram-negative bacteria, serve as pivotal initial triggers [6]. They activate innate immune receptors on host immune cells, cardiomyocytes, endothelial cells, and other cell types, eliciting a robust systemic inflammatory response that generates abundant pro-inflammatory mediators [7,8]. As tissue damage progresses, endogenous damage-associated molecular patterns (DAMPs) are abundantly released [9]. These DAMPs synergize with PAMPs to further amplify inflammatory signaling [9], and induce significant cardiac dysfunction through both direct actions on cardiomyocytes−such as stimulating production of myocardial depressant factors (including TNF-α and IL-1β) and impairment of the cardiac microenvironment [10]. Importantly, injured cardiomyocytes themselves become a source of new DAMPs, thereby establishing a self-perpetuating cycle of persistent inflammatory stimulation and progressive myocardial injury [11]. Despite advances in modern critical care, specific therapies for SIMD remain scarce. Current management focuses on source control, fluid resuscitation, vasoactive agents, and selected anti-inflammatory strategies [12]. While these can partially stabilize hemodynamics and restore cardiac function, they fail to effectively reverse myocardial injury progression or significantly improve long-term prognosis. Many experimental or clinical cardioprotective agents demonstrate suboptimal efficacy. Thus, in−depth dissection of SIMD pathogenesis, identification of key druggable targets, and development of highly effective and safe therapeutics constitute an urgent unmet clinical and scientific need.

In recent years, monomers or extracts derived from traditional Chinese medicine (TCM) have demonstrated distinct advantages in modulating cardiovascular pathologies, garnering increasing recognition within the scientific community [13,14]. Their multi-target and multi−pathway properties hold substantial promise for regulating core pathological processes−including inflammation, oxidative stress, apoptosis, and metabolic dysregulation [15]. This polypharmacological approach stands in contrast to conventional single-target therapies, enabling simultaneous intervention across interconnected pathological axes that drive disease progression. Systematic investigation of TCM constituents not only facilitates novel therapeutic discovery but also provides important clues for understanding disease pathogenesis and identifying new targets [16].

Artemisia carvifolia has been historically employed in traditional medicine to treat “toxic−heat” syndrome, wherein “toxicity” conceptually equates to contemporary inflammatory pathology. Modern phytochemical studies confirm that this syndrome reflects systemic hyperinflammation driven by cytokine storms and oxidative burst. As the core anti−inflammatory constituent identified in this herb, Jaceosidin (JAC) is a natural flavonoid compound with demonstrable biological activities. Contemporary pharmacological research reveals that JAC exhibits broad−spectrum anti-inflammatory, antioxidant, antitumor, and antifibrotic effects. Studies demonstrate that JAC exerts cardioprotective effects by significantly ameliorating myocardial oxidative stress, suppressing inflammatory responses, and reducing cardiomyocyte apoptosis [17]. These findings suggest JAC’s potential value in mitigating inflammatory myocardial injury; however, its specific role in SIMD and underlying molecular mechanisms-particularly whether it involves emerging epigenetic regulatory mechanisms-remain elusive.

Accumulating evidence indicates that severe metabolic derangements during sepsis-particularly dysregulated glycolysis and lactate accumulation-represent not merely manifestations of energy imbalance but may directly participate in gene expression regulation and organ injury through modulating epigenetic modifications [3,18,19]. Mounting evidence highlights the crosstalk between metabolic reprogramming and newly emerging epigenetic regulation across diverse diseases, with lactylation-based modifications playing a pivotal role in inflammatory and cardiovascular pathologies [20,21]. Building on this paradigm, our integrated transcriptomic and metabolomic profiling revealed profound glycolytic pathway reprogramming in TNF-α and LPS induced septic cardiomyopathy models, accompanied by significant elevations of key metabolites lactate and acetate in both in vitro and in vivo systems. Importantly, lactate and acetate serve as essential substrates for lactylation, linking metabolic reprogramming to epigenetic alterations. Mechanistic investigations revealed that JAC’s cardioprotective effects are centrally linked to its potent suppression of sepsis-induced lactylation. This study therefore delineates JAC−mediated regulation of the metabolic and epigenetic axis in SIMD, which likely represents the pivotal mechanism underlying its myocardial preservation.

## 2. Results

### 2.1. In Vitro Protective Effects by JAC Against TNF-α-Induced Injury in AC16 Cells

**Figure 1:**
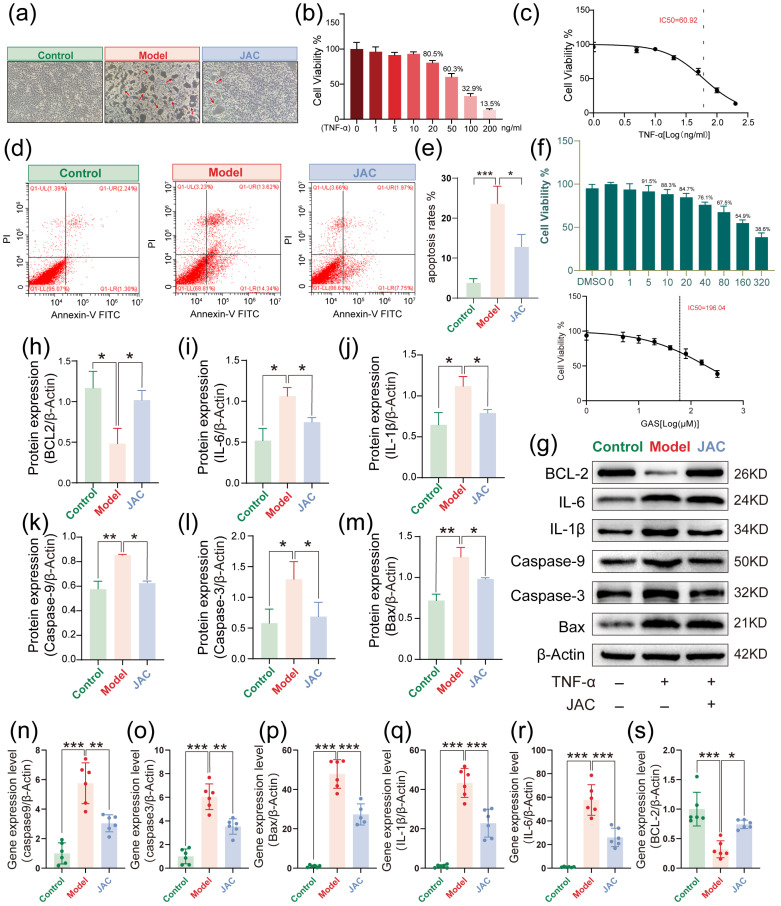
Protective effect of JAC against TNF-α-induced myocardial injury in AC16 cells. (**a**) Representative images of AC16 cardiomyocytes under different conditions, with red arrows indicating cell damage. (**b**) Cell viability of AC16 cells treated with different concentrations of TNF-α for 24 h, *n* = 3. (**c**) IC_50_ values of TNF-α induced AC16 cell model, IC_50_ = 60.92, *n* = 3. (**d**,**e**) Detection of apoptosis levels in the AC16 cells by flow cytometry with a statistical graph, *n* = 3. Each red dot represents a cell. (**f**) Assessment of medication toxicity in JAC cells using the CCK-8 assay, *n* = 3 (**g**–**m**) Western blotting analysis of inflammatory and apoptotic protein expression in AC16 cells following JAC treatment, with statistical graphs, *n* = 3. (**n**–**s**) PCR assay Changes in the expression levels of inflammation and apoptosis-related mRNAs in AC16 cells after administration of JAC and their statistical graphs, *n* = 3. Green: Control Group; Red: Model Group; Blue: JAC Group. Data are presented as mean ± SEM. Statistical significance is shown as * *p* < 0.05, ** *p* < 0.005, *** *p* < 0.001.

JAC treatment demonstrated significant protection against TNF-α−induced cardiomyocyte injury in AC16 cells. Morphological analysis revealed severe cellular damage in TNF-α−treated groups, characterized by membrane rupture and cytosolic shrinkage (Figure 1a), whereas JAC treatment preserved normal cell architecture. CCK−8 assays indicated that 20 ng/mL TNF-α reduced cell viability, while 50 ng/mL treatment caused more pronounced reduction, confirming that 20 ng/mL was principally manifested in TNF-α−induced cardiomyocyte injury rather than nonspecific drug toxicity, with an IC_50_ of 60.92 ng/mL (Figure 1b,c). Flow cytometry detected apoptosis levels in AC16 cells. The results indicated apoptosis in the majority of cardiomyocytes at both early and late stages. JAC treatment significantly attenuated TNF-α−induced apoptosis (Figure 1d,e). The CCK−8 assay demonstrated that within the tested concentration range (particularly at the 20 μM JAC concentration used in this study), JAC alone did not exhibit any significant toxic effects on AC16 cells, with no statistically significant difference in cell viability compared to the control group, and its half-maximal inhibitory concentration (IC_50_) was 196.04 μM. (Figure 1f). Western blotting analysis further revealed that JAC treatment modulated expression of inflammatory and apoptotic proteins, indicating anti-inflammatory and anti−apoptotic effects (Figure 1g–m). Additionally, PCR assays showed altered expression levels of inflammation- and apoptosis−related gene expression after JAC administration, further evidencing its protective mechanism (Figure 1n–s). Collectively, these results demonstrate that JAC effectively protects AC16 cardiomyocytes against TNF-α−induced injury through regulation of inflammatory and apoptotic pathways.

### 2.2. Gene Expression Profiles of mRNA Sequences in JAC-Treated Cells Under TNF-α Induction

**Figure 2:**
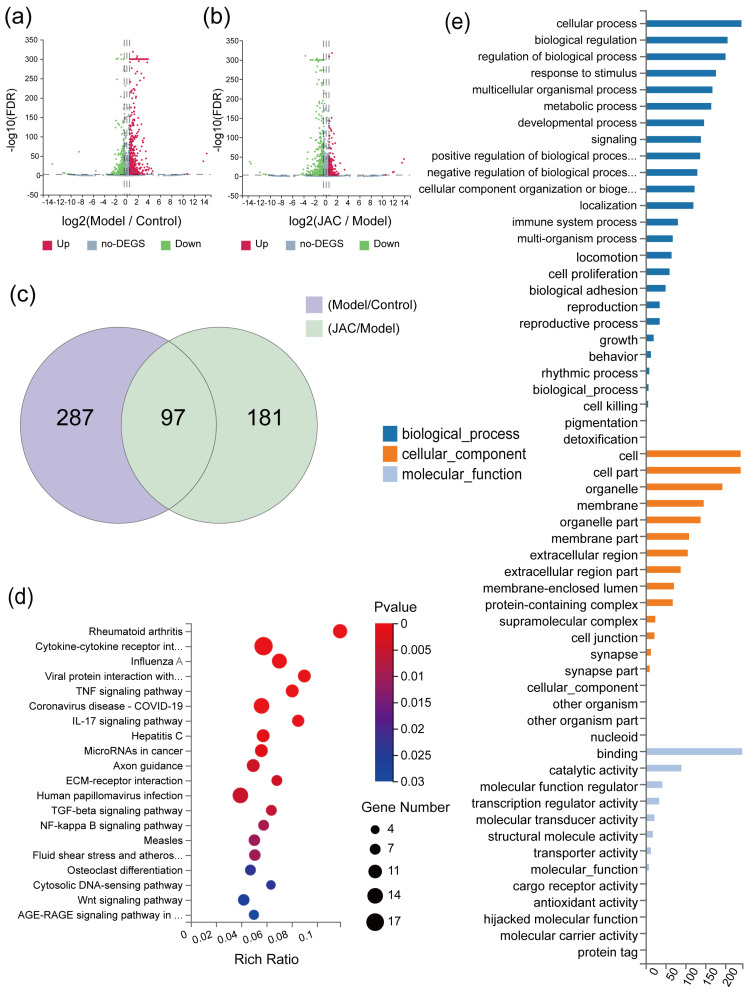
Gene expression profiles of JAC-treated mRNA sequences under TNF-α−inducing conditions. (**a**,**b**) Volcano map of DEGs in Model versus Control (Model vs. Control) and JAC versus Model (JAC vs. Model), jlog2 (fold change) (FC) j > 1 and false discovery rate (FDR) < 0.001 was considered as DEGs. (**c**) Venn interaction of DEGs of Mod−el/Control and JAC/Model. (**d**) KEGG pathway enrichment; a lower Q value indicates a higher degree of enrichment (**e**) GO enrichment of the common DEGs, including biological process, cellular component and molecular functions.

Transcriptome profiling of TNF-α−stimulated cells treated with JAC identified DEGs. A volcano map was constructed to visualize the DEGs between the Model vs. Control (Model vs. Control) and JAC vs. Model (JAC vs. Model) groups, with criteria of |log2 (fold change) (FC)| > 1 and false discovery rate (FDR) < 0.001 (Figure 2a,b). The Venn diagram revealed the intersection of DEGs from both comparisons, indicating the common genes affected by TNF-α induction and JAC treatment (Figure 2c). KEGG pathway enrichment analysis was performed to elucidate the biological pathways associated with these DEGs, with lower Q values suggesting higher degrees of enrichment (Figure 2d). Additionally, GO enrichment analysis was conducted to categorize the common DEGs into biological processes, cellular components, and molecular functions (Figure 2e). These analyses collectively provided elucidating the molecular mechanisms underpinning JAC’s therapeutic effects in modulating gene expression in response to TNF-α−induced myocardial injury.

### 2.3. JAC Remodels the Cellular Metabolic Profile and Alleviates Metabolic Dysfunction Through Gly-Colysis Regulation

**Figure 3:**
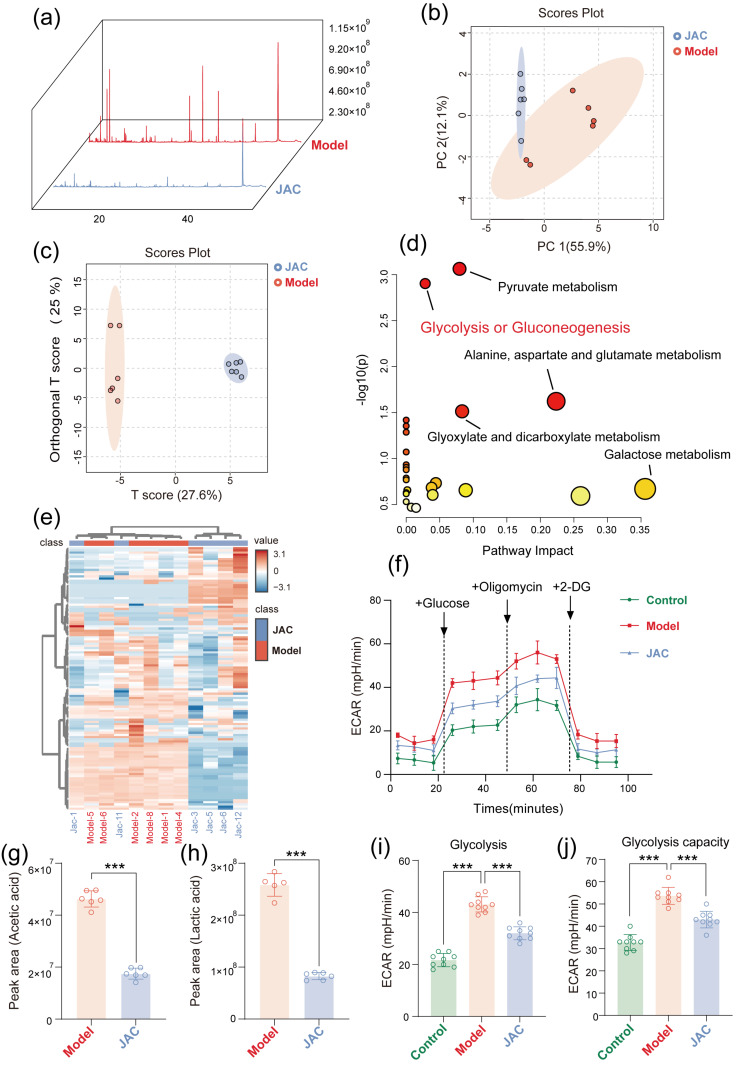
Metabolomic analysis of AC16 cells in TNF-α induced and JAC treated samples. (**a**) Total ion flow plots of cellular metabolites. (**b**) Principal component analysis (PCA) diagram, (**c**) orthogonal partial least squares discrimination analysis diagram (OPLS−DA). (**d**) Pathway enrichment for Pyruvate metabolism, Glycolysis or Gluco-neogenesis, and Alanine, aspartate and glutamate metabolism (VIP > 1). (**e**) Heatmap of all Differently expressed metabolites (DEMs) in the JAC−treated group versus the model group. (**g**,**h**) Top differential metabolite analysis including acetic acid and lactic acid, versus Model (6 vs. 6). (**f**) Extracellular acidification rate (ECAR), (**i**,**j**) Glycolysis detection and Glycolytic capacity measurement statistical plots (9 vs. 9). Data are presented as mean ± SEM. Statistical significance is shown as *** *p* < 0.001.

To fully elucidate the molecular mechanisms of JAC’s metabolic regulation, we performed metabolomic analysis. Metabolomic profiling revealed profound alterations in cellular metabolites upon TNF-α induction and JAC treatment. The total ion flow plots provided an overview of the metabolic landscape (Figure 3a). PCA and OPLS-DA diagrams illustrated the distinct metabolic clustering between groups, reflecting the metabolic shifts induced by JAC (Figure 3b,c). Pathway enrichment analysis pinpointed key metabolic pathways, such as pyruvate metabolism and glycolysis/gluconeogenesis, with VIP > 1 indicating significant metabolic perturbations (Figure 3d). A heatmap imaged the differentially expressed metabolites (DEMs) between the JAC−treated and the Model (Figure 3e). Notably, JAC significantly suppressed the levels of acetic acid and lactic acid, as revealed by top differential metabolite analysis (Figure 3g,h). ECAR measurements and glycolysis detection further corroborated JAC’s impact on glycolytic activity and capacity (Figure 3f,i,j).

### 2.4. JAC’s Specific Inhibition of H3K18la and Its Impact on Metabolic-Epigenetic Coupling

**Figure 4:**
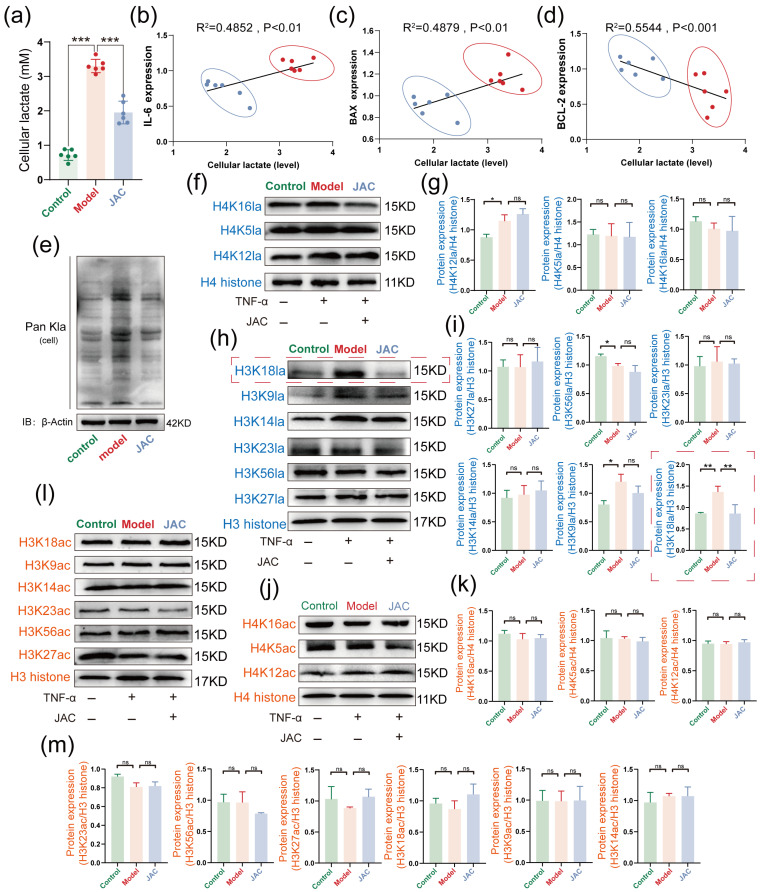
JAC specifically inhibits H3K18la, thereby disrupting the metabolic−epigenetic cou−pling of SIMD. Green: Control Group; Red: Model Group; Blue: JAC Group. (**a**) Intracellular lactate levels in AC16 cells, *n* = 6. (**b**–**d**) Correlation analysis of IL-6, BAX and BCL-2 levels with lactate levels (Model and JAC) in cells and corresponding R^2^ and *p* values, *n* = 6. (**e**) Western blotting screening of Pan Kla expression levels in cells. (**f**,**g**) H4 histone lactylation sites: Western blotting plot showing the lactylation of different histone H4 sites (H4K16la, H4K5la, H4K12la) and their statis−tical plots, *n* = 3. (**h**,**i**) H3 histone lactylation sites: Western blotting plot showing the lactylation of different histone H3 sites (H3K18la, H3K9la, H3K14la, H3K23la, H3K56la, H3K27la) and their statistical plots, *n* = 3. The red dashed box shows the protein expression levels of H3K18la and their statistical plots. (**j**,**k**) H4 histone acetylation sites: Western blotting plot showing the acetylation of different histone H4 sites (H4K16ac, H4K5ac, H4K12ac) and their statistical plots, *n* = 3. (**l**,**m**) H3 histone acetylation sites: Western blotting plot showing the acetylation of different histone H3 sites (H3K18ac, H3K9ac, H3K14ac, H3K23ac, H3K56ac, H3K27ac) and their statistical plots, *n* = 3. Data are presented as mean ± SEM. Statistical significance is shown as * *p* < 0.05, ** *p* < 0.005, *** *p* < 0.001 and ns indicates no significance.

Our investigation delved into the epigenetic mechanisms by which JAC exerts its effects. We found that JAC specifically inhibited intracellular lactate levels in AC16 cells (Figure 4a). Correlation analyses unveiled significant associations between lactate levels and the expression of IL-6, BAX, and BCL-2, with corresponding R^2^ and *p* values underscoring these relationships (Figure 4b,d). Western blotting analyses screened pan lactylation expression levels and revealed that JAC selectively attenuated H3K18la without significantly affecting H3 and H4 histone acetylation sites (Figure 4e–m). These findings implicated H3K18la as a critical epigenetic modification targeted by JAC in the context of myocardial injury.

### 2.5. JAC Regulates SIMD Through SIRT2 Activation and H3K18la Inhibition

**Figure 5:**
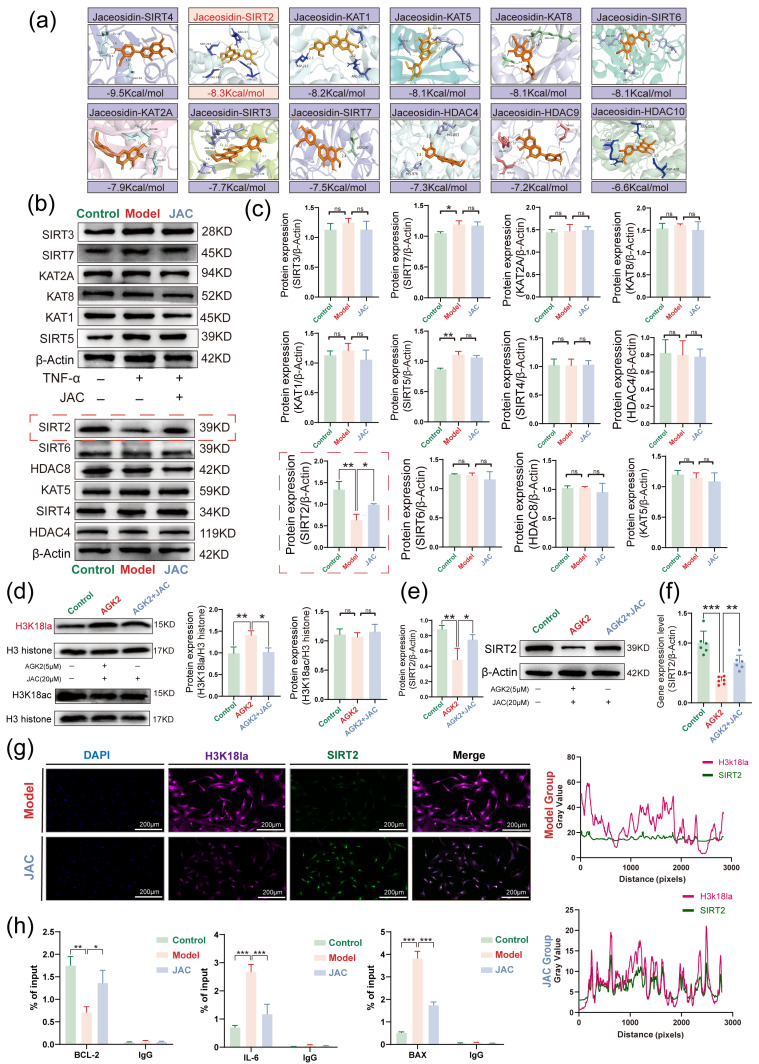
JAC attenuates SIMD by suppressing H3K18la through SIRT2 activation. (**a**) Molecular Docking: Shows the molecular docking results of JAC with a variety of enzymes related to lactoylation including the binding energy (in kcal/mol), the top three highest JAC binding energies were shown to be SIRT4, SIRT2 and KAT1. (**b**,**c**) Western Blotting screening of the expression of lactylation-modifying enzymes associated with SIMD and their statistical plots, *n* = 3. The red dashed box shows the protein expression levels of SIRT2 and their statistical plots. Western Blotting screening of the effect of SIRT2 inhibitor AGK2 (5μM) on: (**d**) H3K18la and H3K18ac. (**e**) SIRT2 expression and their statistical plots, *n* = 3. (**f**) The effect of SIRT2 inhibitor AGK2 (5μM) on the mRNA level of SIRT2 was detected by PCR, *n* = 6. Green: Control Group; Red: AGK2 Group; Blue: AGK2+JAC Group. (**g**) IF staining showed the expression of SIRT2 and H3K18la was mainly located in the nucleus, *n* = 3. (**h**) ChIP−PCR analyses of BCL-2 IL-6 and BAX via reacting with immunoprecipitation of H3K18La antibodies, *n* = 3. Data are presented as mean ± SEM. Statistical significance is shown as * *p* < 0.05, ** *p* < 0.005, *** *p* < 0.001 and ns indicates no significance.

Molecular docking studies demonstrated that JAC exhibited optimal binding energy with SIRT2, suggesting a potential interaction (Figure 5a). Western blotting analyses confirmed that JAC treatment restored SIRT2 expression levels, which were diminished in the modeling process (Figure 5b,c). The introduction of the SIRT2 inhibitor AGK2 reversed JAC’s effects on H3K18la and SIRT2 expression, further validating the involvement of SIRT2 (Figure 5d,e). qPCR analysis also revealed that AGK2 impacted SIRT2 mRNA levels (Figure 5f). IF staining localized SIRT2 and H3K18la expression predominantly to the nucleus (Figure 5g). ChIP-PCR analyses provided evidence of direct regulatory relationships between the H3K18 site and genes such as BCL-2, IL-6, and BAX (Figure 5h). Overall, our results demonstrate that JAC activates SIRT2 to inhibit H3K18la, thereby attenuating SIMD.

### 2.6. JAC Rescues Cardiac Function and Mitigates Myocardial Injury in LPS-Induced In Vivo SIMD in Mice

**Figure 6:**
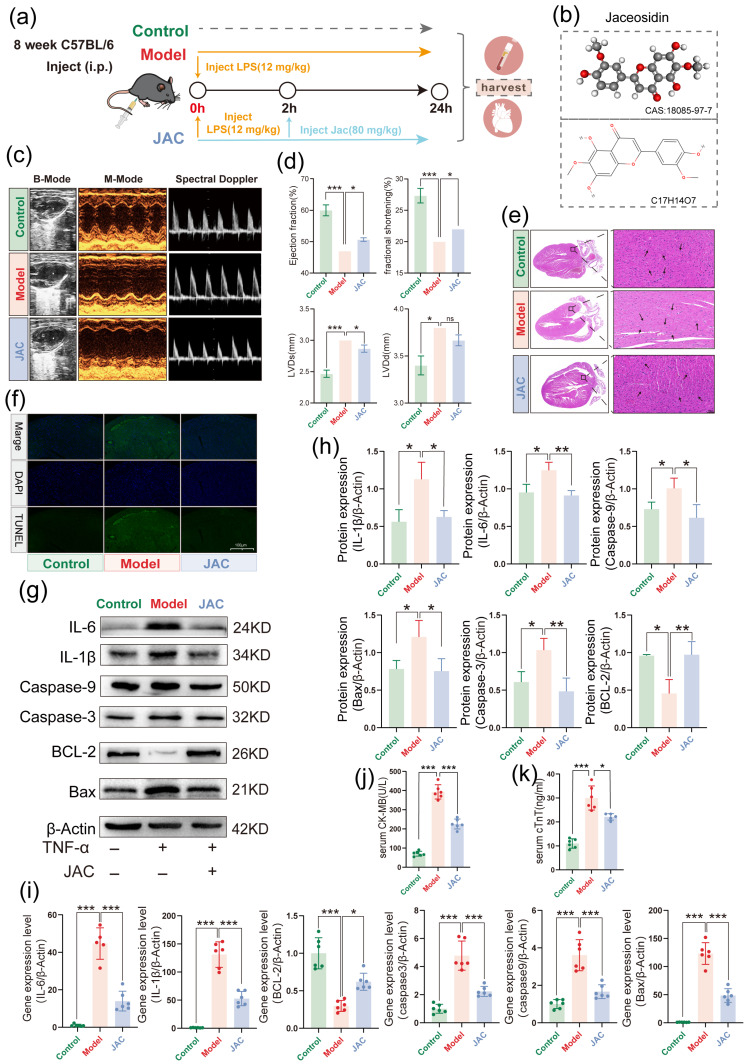
JAC rescues cardiac function and attenuates myocardial injury in LPS-induced SIMD. (**a**) Modeling flowchart: It shows the grouping and treatment process of C57BL/6 mice, including the Control group, the Model group (treated with LPS), and the JAC treatment group (treated with LPS + JAC). (**b**) The structural formula of JAC: It presents the chemical structural formula of Jaceosidin along with its CAS number (18085–97–7), indicating that its molecular formula is C_17_H_14_O_7_. (**c**) Mouse echocardiography: Cardiac ultrasound images of different groups of mice are presented, including M-mode echocardiography to assess cardiac function, *n* = 3. (**d**) Cardiac function index analysis: The histogram showed the LVEF, LVFS, LVDd and LVDs of mice in each group, reflecting the protective effect of JAC on cardiac function, *n* = 3. (**e**) HE showed the pathological morphological changes of myocardial tissue of mice, showing that Jac treatment attenuated LPS–induced myocardial injury. The arrow indicates the specific site of injury. (**f**) TUNEL staining (**g**,**h**) Western Blotting to detect the expression levels of inflammation and apoptosis related proteins in cardiac tissues of SIMD mice and their statistical graphs, *n* = 3. (**i**) PCR assay inflammation and apoptosis–related mRNA expression levels in heart tissues of SIMD mice and their statistical graphs, *n* = 3. (**j**,**k**) cTnT, CK-MB concentrations of cardiac markers in mouse serum were determined by ELISA, *n* = 6. Green: Control Group; Red: Model Group; Blue: JAC Group. Data are presented as mean ± SEM. Statistical significance is shown as * *p* < 0.05, ** *p* < 0.005, *** *p* < 0.001 and ns indicates no significance.

In the in vivo component of our study, we employed a murine model of LPS−induced SIMD to evaluate JAC’s efficacy. The experimental design encompassed Control, Model (LPS−treated), and JAC treatment groups (Figure 6a). The chemical structure of JAC was presented, highlighting its molecular formula (C_17_H_14_O_7_) and CAS number (18085−97−7) (Figure 6b). Echocardiographic assessments, including M-mode imaging, illustrated that JAC preserved cardiac function, as evidenced by improved LVEF and LVFS, and reduced LVDd and LVDs values (Figure 6c,d). Histopathological examination via HE staining revealed that JAC mitigated LPS−induced myocardial injury (Figure 6e). TUNEL staining revealed significantly decreased apoptotic cell populations within myocardial tissues of JAC−treated mice compared to the SIMD model group (Figure 6f). Subsequent immunoblotting and PCR analyses confirmed attenuated expression of inflammatory mediators and apoptosis-associated markers in cardiac specimens from JAC−administered animals (Figure 6g–i). Consistent with these findings, serum quantification of biomarkers of cardiac injury cTnT and CK−MB demonstrated markedly lowered concentrations following JAC intervention (Figure 6j,k).

### 2.7. JAC Alleviates SIMD In Vivo by Modulating Lactate Metabolism and SIRT2-H3K18la Axis

**Figure 7:**
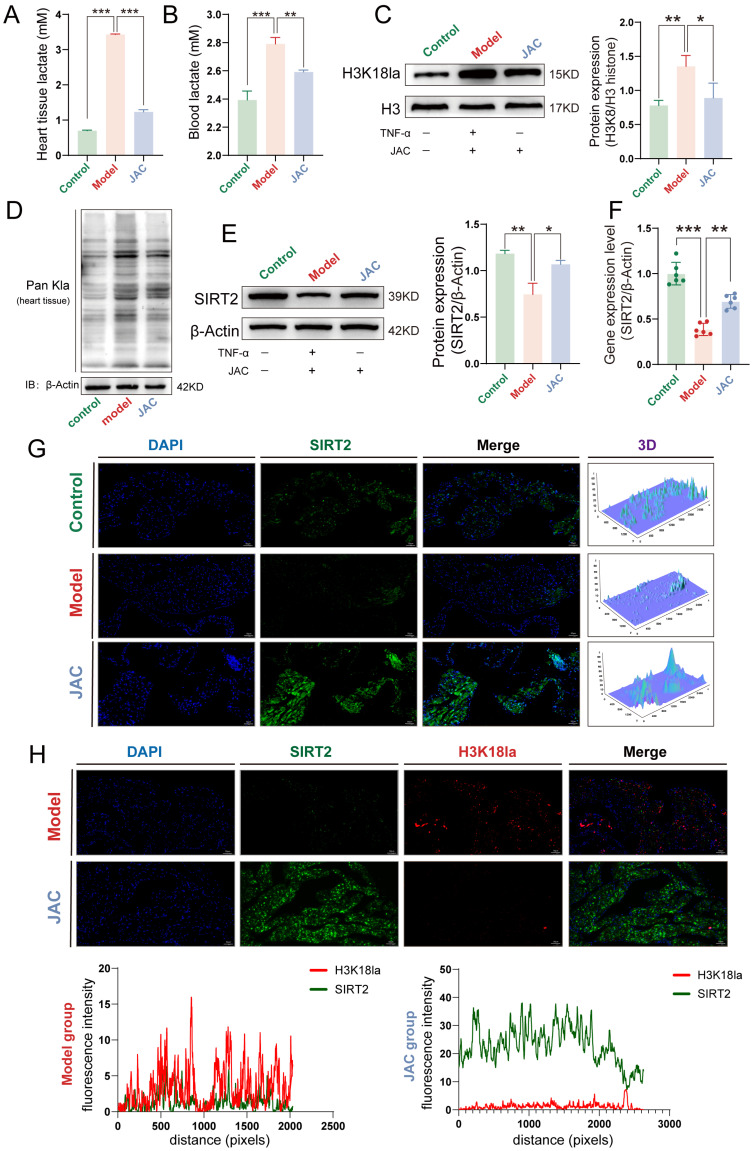
In Vivo modulation of lactate metabolism and SIRT2–H3K18la axis by JAC in SIMD. (**A**) Heart tissue lactate levels in Control, Model, and JAC–treated mice, *n* = 6. (**B**) Blood lactate levels in Control, Model, and JAC-treated mice, *n* = 6. (**C**) Western blotting analysis protein expression levels of H3K18la relative to H3 in cardiac tissue and their statistical graphs, *n* = 3. (**D**) Western blotting analysis Pan lactylation analysis in cardiac tissue. (**E**) Western Blotting to detect the expression levels of SIRT2 in cardiac tissues of SIMD mice and their statistical graphs, *n* = 3. Data are presented as mean ± SEM. (**F**) Changes of SIRT2 mRNA expression levels and their statistical plots, *n* = 6. Green: Control Group; Red: Model Group; Blue: JAC Group. (**G**) Representative immunofluorescence (IF) staining of SIRT2 (green) in cardiac tissue sections from the transverse mid-ventricular plane. Nuclei are counterstained with DAPI (blue). The lower panel shows a 3D reconstructed image for quantitative analysis. Scale bar = 100 μm, *n* = 3. (**H**) Representative IF co-staining of SIRT2 (green) and H3K18la (red) in cardiac tissue sections from the transverse mid-ventricular plane, showing their co-localization in cardiomyocyte nuclei (yellow in merge). Scale bar = 100 μm, *n* = 3 (Representative histological sections from the transverse plane at the mid-ventricular level are shown. The insets highlight characteristic pathological changes within the left ventricular myocardium). Data are presented as mean ± SEM. Statistical significance is shown as * *p* < 0.05, ** *p* < 0.005, *** *p* < 0.001.

In our in vivo validation studies, we Demonstrated a Considerable reduction in lactate levels in both cardiac tissue and blood serum following JAC treatment. Specifically, heart tissue lactate quantification revealed pronounced reductions in the JAC intervention group (Figure 7A). Similarly, blood lactate levels exhibited a pronounced reduction, further corroborating the lactate-lowering effects of JAC (Figure 7B). Western blotting analysis of cardiac tissue revealed a significant suppression of H3K18la in JAC–treated mice (Figure 7C). This result aligns with our in vitro observations, reinforcing the consistency of JAC’s mechanism in targeting H3K18la across experimental models. Pan lactylation analysis demonstrated a broad reduction in lactylation across cardiac proteins in JAC–treated mice (Figure 7D). Western blotting showed that JAC treatment restored SIRT2 expression levels, which were downregulated in the LPS–induced model group (Figure 7E), and the PCR results similarly confirmed this conclusion (Figure 7F). To further investigate the expression of SIRT2 in cardiac tissues, we performed IF staining on cardiac tissue sections. IF imaging demonstrated increased nuclear expression of SIRT2 following JAC treatment (Figure 7G), with co–localization analysis confirming that both SIRT2 and H3K18la co–localized within cardiomyocyte nuclei (Figure 7H). The in vivo results were highly consistent with our in vitro findings, confirming that JAC inhibits SIMD in vitro and in vivo by promoting SIRT2-mediated lactylation of histone H3K18.

## 3. Discussion

JAC, a naturally derived bioactive compound, has demonstrated therapeutic potential across multiple disease models. Previous studies revealed that JAC significantly suppresses LPS induced pulmonary inflammation while mitigating oxidative stress and tissue damage [22]. In metabolic regulation, this compound reduces oxidative stress and inflammation by activating insulin signaling pathways, effectively ameliorating metabolic dysregulation and organ injury [23]. In osteoarthritis models, JAC inhibits NF–κB signaling transduction through blocking IκB degradation, thereby attenuating inflammatory responses and retarding cartilage degeneration [24]. Furthermore, JAC exhibits potent metabolic modulatory effects in obesity models: reducing body weight, blood glucose, and insulin levels; improving glucose tolerance and insulin sensitivity; concurrently decreasing serum triglycerides and cholesterol. Its hepatoprotective mechanism involves suppressing hepatic lipid accumulation, downregulating lipogenic gene expression, and improving liver function parameters [25]. In cardiovascular protection, JAC operates through dual pathways [26]: activating the nuclear factor Nrf2 pathway to enhance antioxidant enzyme expression, reduce ROS and lipid peroxidation, thereby alleviating cardiomyocyte oxidative stress and apoptosis; while concurrently inhibiting NF–κB mediated inflammatory cascades to reduce proinflammatory cytokine levels and mitigate cardiac injury. This study uncovers a novel cardioprotective mechanism of JAC in SIMD, featuring specific targeting of the metabolic epigenetic axis through upregulating deacetylase SIRT2 to selectively suppress H3K18la. Compared to conventional broad spectrum anti-inflammatory agents, JAC demonstrates superior therapeutic advantages by precisely modulating sepsis associated metabolic derangements and epigenetic dysregulation.

During sepsis, significant alterations occur in the glycolytic pathway within both cardiomyocytes and macrophages [27]. PAMPs activate TLR4 signaling, triggering p38 MAPK pathway activation. This upregulates the key glycolytic regulator PFKFB3, driving accelerated glycolytic flux, which is critically associated with myocardial dysfunction. Notably, this metabolic perturbation instigates a pathogenic cascade wherein pathogen invasion activates the innate immune response, exacerbating inflammation and apoptosis in cardiomyocytes while concurrently disrupting their metabolic and functional integrity. The substantial release of inflammatory and pro-apoptotic factors induces a metabolic shift towards glycolysis in both cardiomyocytes and macrophages, resulting in lactate accumulation. Beyond its role as a metabolic byproduct of glycolysis, lactate serves important functions in modulating processes such as T helper cell and macrophage regulation [28]. Furthermore, lactate actively participates in regulating histone lactylation [20]. This multifaceted role of lactate provides a novel perspective for in–depth mechanistic analysis of the interplay between metabolic and epigenetic regulation in SIMD. In our study, profound alterations in the glycolytic pathway were observed in TNF-α and LPS–induced models of septic cardiomyopathy, characterized by significant elevations in key metabolites lactate and acetate. These findings align with previous reports emphasizing the central role of dysregulated glycolytic flux in the pathophysiology of sepsis. Dysregulation of metabolic pathways not only disrupts energy homeostasis within cardiomyocytes but also acts as a critical driver of inflammatory signaling and oxidative stress, thereby further exacerbating myocardial injury. By targeting metabolic reprogramming, JAC offers a novel therapeutic strategy to disrupt the vicious cycle of metabolic dysfunction and inflammation characteristic of SIMD.

Lactate is not only a metabolic waste product and prognostic marker in sepsis, but also a key regulatory molecule mediating immune imbalance and organ injury. Its pathological accumulation drives multi–organ injury by inducing histone lactylation [19]. On one hand, in sepsis-associated acute lung injury (ALI), p300–mediated H3K18la directly upregulates the methyltransferase METTL3, activating the ACSL4-dependent ferroptosis pathway leading to alveolar epithelial cell injury [29]. On the other hand, H3K18la levels positively correlate with disease severity [30], and targeted intervention can significantly improve survival rates. This study found that under pathological conditions induced by TNF-α/LPS, lactate accumulation driven by glycolytic disruption specifically promotes an increase in H3K18la, while H3K18ac remains stable, suggesting that lactate preferentially occupies modification sites and drives lactylation in the metabolic stress environment of SIMD. SIRT2, as a key delactylase, possesses structural adaptability in its substrate-binding domain for the lactyl group (long–chain fatty acyl group), conferring selective catalytic advantages [31]. Research shows that SIRT2 specifically inhibits the cuproptosis pathway by regulating METTL16 K229 lactylation [32]. Notably, SIRT2 prioritizes the processing of lactylation at the expense of its acetyl group removal capability, thereby avoiding perturbation of the physiological acetylation network. Based on this, this study found that unlike broad–spectrum epigenetic modulators, under inflammatory stress, JAC precisely and preferentially targets H3K18la by activating SIRT2 without disrupting the physiological acetylation network. This specificity avoids potential off–target effects, highlighting its therapeutic advantage for septic myocardial injury.

Lactylation, as a key intersection of metabolism and epigenetics, exhibits dual protective and pathogenic roles in cardiovascular diseases by regulating inflammatory balance, energy metabolism, and cellular remodeling [33,34]. Studies show that the extracellular matrix protein Lumican significantly promotes histone lactylation at H3K14 and H3K9 sites by enhancing glycolysis and lactate production, thereby driving the progression of aortic valve calcification lesions [13]. Conversely, targeted inhibition of the key lactylation enzyme p300 effectively reduces H3 histone lactylation levels and alleviates valvular calcification injury [20]. In atherosclerosis, lactylation improves plaque stability and the inflammatory microenvironment by inhibiting RUNX1 transcriptional activity and enhancing chromatin accessibility [21]. Notably, lactate accumulation in the myocardial hypoxic microenvironment triggers histone lactylation, activating the “lactate clock” mechanism to precisely coordinate anti-inflammatory responses and tissue repair signaling networks [35]. Our research indicates that in vitro, TNF-α stimulation activates the glycolytic pathway, leading to lactate accumulation, which subsequently drives H3K18la, exacerbating cardiomyocyte inflammation and apoptosis. JAC, by upregulating SIRT2 expression, specifically targets H3K18la, effectively reversing cardiomyocyte damage and alleviating SIMD. In vivo experiments similarly confirmed the protective effects of JAC, as it significantly improved cardiac function in a septic mouse model, reduced myocardial injury, and lowered levels of inflammation and apoptosis markers. These findings suggest JAC as a potential therapeutic agent that may offer new strategies for the clinical treatment of SIMD.

We comprehensively assessed the pharmacological effects of JAC on SIMD and mechanistically confirmed its functional involvement in a metabolic-epigenetic regulatory pathway through integrated in vivo and in vitro models. Our investigation revealed that lactate accumulation during SIMD drives pathological reprogramming of histone lactylation, with H3K18 identified as the pivotal modification site via Western blot screening. Significantly, JAC alleviated SIMD by modulating SIRT2–dependent delactylation to inhibit H3K18la, consequently suppressing transcriptional activation of pro-inflammatory IL-6 and pro–apoptotic BAX genes while restoring BCL-2 expression. This study establishes a novel mechanistic framework for JAC’s cardiovascular protection, positioning it as a promising therapeutic candidate for SIMD through targeting the newly identified SIRT2–H3K18la regulatory axis.

This study systematically reveals a novel mechanism by which JAC improves SIMD through the SIRT2–H3K18la axis, but it has certain limitations. Although the experimental models primarily employed TNF-α/LPS–induced AC16 cells and murine sepsis systems, these platforms inadequately recapitulate sepsis–specific immune-cardiomyocyte crosstalk, particularly the complex interaction networks between immune cells and cardiomyocytes. Future research should establish immune cell-cardiomyocyte co–culture systems or animal models to enhance pathological simulation comprehensiveness. Mechanistically, although AGK2 inhibition experiments confirmed the necessity of SIRT2, no cardiomyocyte specific SIRT2 gene knockout animal model was used to further validate its cardiac specific role. Subsequent studies could construct conditional knockout models to clarify SIRT2’s cell autonomous function. Additionally, while this study mainly focused on H3K18la regulation of BCL-2, BAX, and IL-6, histone lactylation may affect broader gene networks, such as metabolic reprogramming related genes. Future work could employ ChIP–seq technology to comprehensively map the genome-wide binding profile of H3K18la for deeper mechanistic understanding. Furthermore, the pharmacokinetic properties of JAC (e.g., cardiac tissue distribution, metabolic stability) remain unevaluated, which are critical unresolved issues for clinical translation planning. These limitations provide clear directions for future research, but the core conclusions of this study remain reliable, offering important theoretical foundations and experimental support for treating septic myocardial injury.

## 4. Materials and Methods

### 4.1. Cell Culture Protocols and Experimental Treatments

AC16 cells (Human-derived cardiomyocytes, NO: CL–0790, Procell Life Science & Technology Co., Ltd., Wuhan, China) were cultured in Minimum Essential Medium (MEM, NO: PM150411, Procell Life Science & Technology Co., Ltd.) supplemented with 10% fetal bovine serum (FBS; Cell Box, Changsha, China) under standard conditions (37 °C, 5% CO_2_). Until the cells reached approximately 80% confluence, cells were subjected to concurrent stimulation with 20 ng/mL TNF-α (PeproTech, Cranbury, NJ, USA, NO: 300-01A) and 20 μM JAC for 24 h [36]. AGK2, a selective SIRT2 inhibitor, suppresses cellular proliferation and growth in a dose-dependent manner while exhibiting no cytotoxicity at low concentrations. To understand more about the role of SIRT2, we treated cells with AGK2 (MedChemExpress, Monmouth Junction, NJ, USA, Cat: HY-100578 CAS No: 304896–28–4). A statement to confirm that all methods were carried out in accordance with relevant guidelines and regulations.

### 4.2. Cell Viability Assay

AC16 cells were seeded in 96–well plates (5 × 10^3^ cells/well) and exposed to designated treatments. Cell viability was assessed using a CCK-8 (Cell Counting Kit-8) assay kit (NO: K1018, APExBIO, Houston, TX, USA) according to manufacturer’s protocol. Cells were incubated with CCK-8 reagent for 2 h at 37 °C, after which absorbance was measured at 450 nm using a microplate reader. Data from three independent biological replicates were normalized to untreated controls and analyzed by one-way ANOVA to determine statistically significant differences (*p* < 0.05) in cell viability across TNF-α or JAC treatment groups.

### 4.3. Flow Cytometric Analysis of Apoptosis

AC16 cells were seeded in 6-well plates and incubated with 20 μM JAC for 24 h. Subsequently, cells were harvested, subjected to Annexin V–FITC/propidium iodide (PI) double staining, and analyzed by flow cytometry.

### 4.4. RNA Sequencing (RNA-Seq)

Changes in cellular mRNA profiles across experimental treatments were investigated through quantitative RNA–seq methodology. Cellular material was harvested with TRIzol reagent (Vazyme, Nanjing, China, NO: R401-01) followed by RNA extraction. Purified RNA samples were subsequently transferred to BGI Ltd. (Wuhan, China) for RNA sequencing. Raw sequencing reads underwent computational processing using R software (version 3.5.1) to determine differentially expressed genes (DEGs). Enrichment analyses of identified DEGs were conducted for Gene Ontology (GO) annotations and KEGG pathway databases [37].

### 4.5. Gas Chromatography-Mass Spectrometer (GC–MS) Metabolome Analysis

AC16 cells were rinsed thrice with ice-cold PBS and lysed in pre–chilled HPLC-grade methanol (−80 °C). Cell lysates underwent three freeze–thaw cycles (liquid nitrogen/37 °C water bath, 10 min per cycle) followed by centrifugation (12,000× *g*, 4 °C, 10 min). The clarified supernatant was subsequently dried under nitrogen gas at 30 °C.

Dried extracts were derivatized with 80 μL methoxyamine hydrochloride (20 mg/mL in pyridine) at 37 °C for 90 min, followed by addition of 80 μL N,O–bis(trimethylsilyl)trifluoroacetamide (BSTFA) with 1% trimethylchlorosilane (TMCS). Samples were vortex–mixed (1 min), centrifuged (10,000× *g*, 1 min), and incubated at 70 °C for 1 h. Derivatized extracts were centrifuged (12,000× *g*, 4 °C, 10 min), and supernatants were transferred to GC vials.

GC–MS analysis was performed within 48 h using a Trace 1300 GC system coupled to ISQ QD mass spectrometer (Thermo Scientific, Waltham, MA, USA) equipped with a TG–5MS capillary column (30 m × 0.25 mm × 0.25 μm). Injection volume: 1 μL in splitless mode. At the final stage of the experiment, data processing was performed using the Chromatographic Fingerprint Similarity Evaluation System (2012 edition) and SIMCA-P+ 14.1 (Umetrics, Umeå, Sweden).

### 4.6. Real-Time Metabolic Flux Analysis Using Seahorse XF Technology

AC16 cell suspensions were seeded at 6 × 10^4^ cells/well in Matrigel™-precoated Seahorse XF96 cell culture microplates (250 μL/well) and incubated for 12 h at 37 °C/5% CO_2_. For drug-treated groups, 20 μM JAC was administered after initial incubation, followed by continued culture for 24 h total. Prior to assay, cells were washed thrice with prewarmed (37 °C) XF assay medium (pH 7.4) and maintained in 500 μL/well of the same medium. Microplates were equilibrated for 60 min in a non–CO_2_ incubator at 37 °C. 10 mM glucose, 1 μM oligomycin and 50 mM 2-DG were added to the cell culture plates. Extracellular acidification rate (ECAR) was measured over 100 min (3 measurement cycles per injection, 5 min mix/2 min wait/3 min measure). Following experimental completion, datasets were processed using Agilent Seahorse Wave analytical software (Seahorse Wave 2.6.1).

### 4.7. Lactate Acid Detection

Cardiac tissues (50 mg) were homogenized in 500 μL ice-cold Lactate Assay Buffer using a Polytron homogenizer. Homogenates were centrifuged at 12,000× *g* (4 °C, 5 min), and supernatants were collected for analysis. For cellular lactate measurement, AC16 cells grown in 6-well plates were trypsinized, washed twice with ice–cold PBS, and resuspended in 200 μL Lactate Assay Buffer per 1 × 10^6^ cells. Cell suspensions underwent ice–bath sonication (200 W, 3 s pulse/7 s interval, 30 cycles; Sonics Vibra–Cell VCX130) followed by centrifugation (12,000× *g*, 4 °C, 5 min).

Supernatants from tissues or cells were assayed using a Lactate Content Detection Kit (NO: KTB1100, ABBkine, Atlanta, GA, USA). Aliquots (50 μL) were incubated with 50 μL assay reagent at 37 °C for 30 min in the dark. Absorbance at 450 nm was measured.

### 4.8. Molecular Docking

The 3D structures of the compounds were retrieved from the PubChem database (https://pubchem.ncbi.nlm.nih.gov/ (accessed on 19 July 2025)). Preprocessing of these structures, including energy minimization and conversion to PDBQT format, was carried out using OpenBabel–2.3.2 and AutoDock4.2.6 tools. The crystal structure of the target protein was obtained from the UniProt database (https://www.uniprot.org/ (accessed on 19 July 2025)). To prepare the protein for docking, dehydration was performed to remove all water molecules, and the native ligand was extracted using PyMOL 2.4.0. The binding energy calculations and molecular docking simulations were performed using AutoDock. The docking process involved an automated search for the optimal binding conformation of each compound within the predefined binding site of the protein. The results were visualized and analyzed using PyMOL’s visualization tools to assess the interaction between the compounds and the target protein.

### 4.9. Immunofluorescence (IF) Assay

AC16 cells were seeded on glass coverslips at 1 × 10^3^ cells/coverslip. After 24 h incubation, cells were washed twice with PBS, fixed with 4% paraformaldehyde for 20 min at room temperature, and permeabilized with 0.2% Triton X–100/PBS for 10 min. Non–specific binding was blocked with 5% bovine serum albumin (BSA)/PBS for 30 min. Samples were incubated overnight at 4 °C with primary antibodies against: SIRT2 (1:200; Immunoway, San Jose, CA, USA) and H3K18la (1:200, Zen-Bio, Durham, NC, USA). After PBS washes, cells were incubated with goat anti-rabbit IgG and goat anti-mouse IgG secondary antibodies for 60 min at 37 °C in the dark. Nuclei were counterstained with 4′,6-diamidino–2–phenylindole for 5 min, and then imaged under microscope.

### 4.10. Chromatin Immunoprecipitation Quantitative Polymerase Chain Reaction (ChIP-qPCR) Assay

Assays were performed utilizing a ChIP Assay Kit (Beyotime, Wuhan, China, NO: P2078) in strict accordance with the manufacturer ’s instructions. In detail, cells were initially subjected to cross–linking with 1% formaldehyde to establish covalent linkages between DNA and associated proteins. Subsequently, the harvested cell lysates underwent sonication to yield chromatin fragments within the size range of 200–300 bp, ensuring optimal accessibility for downstream immunoprecipitation procedures. The prepared chromatin samples were then subjected to immunoprecipitation employing an anti-P65 antibody, with IgG serving as a negative control to assess background nonspecific binding.

In order to evaluate the enrichment of specific genomic regions, qPCR was conducted on the immunoprecipitated DNA samples. The qPCR primers were meticulously designed to specifically amplify the proximal promoter regions of the *IL-6*, *BCL-2* and *BAX* genes, which harbor putative *H3K18la* binding sites. Following quantification, the percent input values derived from each experiment were normalized and converted into fold changes relative to an untreated control group. Ultimately, the average fold changes garnered from three independent experimental replicates were calculated and plotted to present the experimental outcomes in a comparative and quantifiable manner.

ChIP-qPCR primer sequences employed in this study were:

*BAX*: F: 5′-TCGCGATCGGCCTGGTTC-3′, R: 5′-TAAAGGGACAGCAACTGT-3′;

*IL-6*: F: 5′-TGGAACTGCCAGCGGCGG-3′, R: 5′-TCCTTTTTGTCCCCCGGG-3′;

*BCL-2*: F: 5′-TCGAGAGGAGTTATAATA-3′, R: 5′-GTTTTTAGACTTTCTTAT-3′;

### 4.11. Animal Model and Treatment

Male C57BL/6J mice (6–8 weeks old, 22 ± 2 g) were obtained from Hangzhou Ziyuan Experimental Animal Technology Co., Ltd., Hangzhou, China, (License SYXK(E) 2023-0067) and maintained under standardized conditions (24 ± 2 °C, 12 h light/dark cycle). All experimental protocols were approved by the Animal Ethics Committee of Hubei University of Chinese Medicine (Approval No. HUCMS 202311047) and emphasis on minimizing animal suffering and sample sizes. Animals were randomly assigned to three experimental groups (*n* = 6/group): normal control (Control), LPS (Model)–induced model, and JAC-treated. SIMD was induced via i.p. of 12 mg/kg LPS (Sigma, Cream Ridge, NJ, USA, USA, NO: L2880) [38]. Two hours post-modeling, the JAC-treated group received 80 mg/kg JAC (purity ≥ 98%, ChemFaces, Wuhan China, CFN90386; CAS 18085-97-7) dissolved in saline via intraperitoneal injection, while Control groups received equivalent saline volumes. The murine dosage was determined using validated interspecies dose translation (human dose [mg/kg] × 70 kg × 0.0026/0.02 kg) [39,40]. Twenty-four hours after LPS administration, euthanasia was performed by intravenous injection of sodium pentobarbital (100 mg/kg). After euthanasia, the excised hearts were perfused retrogradely via the aorta with ice-cold phosphate-buffered saline (PBS) to remove residual blood. For molecular analyses (Western blotting and qRT–PCR), whole hearts were flash-frozen in liquid nitrogen and pulverized to ensure homogeneity. For histological examinations (H&E, TUNEL) and immunofluorescence (IF), whole hearts were immersion–fixed in 4% paraformaldehyde, followed by standard paraffin embedding. Transverse sectioning was performed at the mid-ventricular level to ensure consistent anatomical orientation and inclusion of the left ventricular free wall, interventricular septum, and right ventricle in all analyses. All histological and IF assessments were conducted on sections obtained from this standardized anatomical plane. Terminal blood collection was executed via cardiac puncture, with plasma isolated following 2 h coagulation at room temperature and centrifugation (3000 rpm, 15 min, 4 °C), then stored at −80 °C. Excised hearts were perfused with ice-cold PBS to remove residual blood, with one portion immersion-fixed in 4% paraformaldehyde for histology and the remainder flash-frozen in liquid nitrogen for molecular analyses at −80 °C. A statement to confirm that all methods were carried out in accordance with relevant guidelines and regulations.

### 4.12. Mouse Echocardiography

Prior to tissue collection, transthoracic echocardiography was performed under isoflurane anesthesia. Following chest hair removal, mice were positioned supine on a temperature–controlled platform (37 °C) to maintain normothermia. A standardized layer of acoustic coupling gel was applied to the precordial region before cardiac imaging using the Vevo 2100 system (FUJIFILM VisualSonics, Inc., Toronto, ON, Canada). Comprehensive evaluation included two-dimensional, M–mode, and color Doppler imaging at the aortic valve level. To ensure objective assessment, operators were blinded to group assignments during image acquisition and analysis. Hemodynamic parameters-specifically left ventricular ejection fraction (LVEF), left ventricular fractional shortening (LVFS), Left ventricular end–diastolic internal diameter (LVDd), left ventricular end–systolic internal diameters (LVDs) and peak aortic flow velocity-were measured across three consecutive cardiac cycles with reported values representing averaged measurements. This protocol evaluated the effects of JAC on cardiac performance in the experimental model.

### 4.13. Histopathological Analysis

Cardiac tissues from mice were fixed in 4% paraformaldehyde, dehydrated through a graded ethanol series, embedded in paraffin, and sectioned at 5 μm thickness. TdT-mediated dUTP nick end labeling (TUNEL) staining and Hematoxylin and eosin (H&E) staining were subsequently performed to assess histopathological changes and early cardiomyocyte apoptosis as a means of evaluating the cardioprotective efficacy of JAC.

### 4.14. Enzyme Linked Immunosorbent Assay (ELISA)

Cardiac tissue and serum specimens were harvested from experimental mice. Serum levels of cardiac injury biomarkers—cardiac troponin T (cTnT) and creatine kinase–MB isoenzyme (CK–MB)—were measured using Elabscience kits (CK–MB: E–EL–M0355; cTnT: E–EL–M1801) per manufacturer’s protocols to evaluate JAC’s protective effects on myocardial tissue.

### 4.15. Western Blotting

For the preparation of protein samples, mice heart tissues were homogenized at 4 °C and collected using RIPA lysis buffer (Servicebio, Wuhan, China, NO: G2002-100ML). Similarly, AC16 cells cultured were harvested by scraping and collected using the same RIPA lysis buffer. The lysates were thoroughly lysed on ice, followed by centrifugation at 12,000× *g* for 10 min at 4 °C. The resulting supernatants were collected and stored at −20 °C until further use. For Western blotting analysis, the proteins were denatured by heating at 100 °C for 10 min. Gel electrophoresis was performed to separate the proteins based on their molecular weights, followed by transferring onto a membrane for immunoblotting. The membrane was incubated with the primary antibody at 4 °C overnight with gentle shaking. After washing the membrane three times with TBST buffer, the HRP–conjugated secondary antibody diluted in TBST was applied, and the membrane was incubated at 37 °C for 1 h. Following additional TBST washes to remove unbound antibodies, the protein bands were visualized using an ECL detection system. The band intensities were quantified and analyzed using Quantity One software V4.6.2.

The antibody for Western blotting used for the experiments are listed in Table 1.

### 4.16. Quantitative Real-Time PCR (qRT–PCR)

Primer sequences were designed using the NCBI Primer-BLAST (https://blast.ncbi.nlm.nih.gov/Blast.cgi (accessed on 23 July 2025)) tool. Total RNA was extracted from mouse heart tissues and AC16 cells, followed by reverse transcription to synthesize cDNA. The target genes were then amplified using a real-time PCR system. The relative mRNA expression levels were calculated using the 2−ΔΔCt method and normalized to the internal control *β-actin*. The primers for the target genes were synthesized by Beijing Qingke Biotechnology Co., Ltd. (Wuhan, China).

The sequences of the primers are listed in Table 2.

### 4.17. Statistical Analysis

Statistical analyses were conducted using SPSS 22.0 and GraphPad Prism 8.0 software. Parametric tests were applied according to experimental design: one–way ANOVA for multiple group comparisons and Student’s *t*-test for pairwise comparisons. Post hoc analyses proceeded only when ANOVA yielded significant F-values (*p* < 0.05) with confirmed variance homogeneity. Results are presented as mean ± standard deviation (SD), with statistical significance defined as *p* < 0.05.

## Figures and Tables

**Table 1:** Antibody for Western Blotting.

Antibody	Cat No	Producer
TNF-α	346654	ZenBio, Chengdu, China
IL-1β	660092	ZenBio, China
IL-6	347023	ZenBio, China
BAX	R380709	ZenBio, China
BCL-2	381702	ZenBio, China
Caspase-9	680095	ZenBio, China
Caspase-3	R22842	ZenBio, China
β-Actin	R51031	ZenBio, China
Pan Kla	PTM-1425	PTM BIO, Hangzhou, China
Anti-L-Lactyl-Histone H4 (Lys16)	PTM-1417RM	PTM BIO, China
Histone H4	PTM-1015RM	PTM BIO, China
Anti-L-Lactyl-Histone H4 (Lys5)	PTM-1409	PTM BIO, China
Anti-L-Lactyl-Histone H4 (Lys12)	PTM-1411	PTM BIO, China
Histone H3	PTM-6613	PTM BIO, China
Anti-L-Lactyl-Histone H3 (Lys14)	PTM-1414RM	PTM BIO, China
Anti-L-Lactyl-Histone H3 (Lys18)	PTM-1406RM	PTM BIO, China
Anti-L-Lactyl-Histone H3 (Lys9)	PTM-1419RM	PTM BIO, China
Anti-L-Lactyl-Histone H3 (Lys23)	PTM-1413RM	PTM BIO, China
Anti-L-Lactyl-Histone H3 (Lys56)	PTM-1421RM	PTM BIO, China
Anti-L-Lactyl-Histone H3 (Lys27)	PTM-1428	PTM BIO, China
Anti-Acetyl-Histone H4 (Lys16)	PTM-122	PTM BIO, China
Anti-Acetyl-Histone H4 (Lys5)	PTM-119	PTM BIO, China
Anti-Acetyl-Histone H4 (Lys12)	PTM-121RM	PTM BIO, China
Anti-Acetyl-Histone H3 (Lys14)	PTM-113RM	PTM BIO, China
Anti-Acetyl-Histone H3 (Lys18)	PTM-114RM	PTM BIO, China
Anti-Acetyl-Histone H3 (Lys9)	PTM-112RM	PTM BIO, China
Anti-Acetyl-Histone H3 (Lys23)	PTM-115RM	PTM BIO, China
Anti-Acetyl-Histone H3 (Lys56)	PTM-162	PTM BIO, China
Anti-Acetyl-Histone H3 (Lys27)	PTM-116RM	PTM BIO, China
SIRT7 Polyclonal antibody	12994-1-AP	Proteintech, Rosemont, IL, USA
SIRT4 Rabbit pAb	862208	ZenBio, China
SIRT2 Rabbit mAb	R25722	ZenBio, China
SIRT6 Rabbit mAb	R381408	ZenBio, China
SIRT3 Rabbit mAb	R25724	ZenBio, China
SIRT5 Rabbit mAb	R381473	ZenBio, China
HDAC8 Rabbit mAb	R381478	ZenBio, China
HDAC4 Rabbit pAb	R381467	ZenBio, China
KAT8 Rabbit mAb	R383056	ZenBio, China
KAT1 Rabbit mAb	R383080	ZenBio, China
KAT5 Mouse mAb	221268	ZenBio, China
KAT2A Rabbit mAb	R24422	ZenBio, China

**Table 2:** Primer sequences for real-time RT-PCR.

Gene	Primer (5′-3′)
*H-IL-1β*	F: AGCACCTTCTTTCCCTTCATCTT R: CACCACTTGTTGCTCCATATCCT
*M-IL-1β*	F: CAGCACATCAACAAGAGCTTCAG R: GAGGATGGGCTCTTCTTCAAAGA
*H-IL-6*	F: AACATGTGTGAAAGCAGCAAAGA R: CTCTGGCTTGTTCCTCACTACTC
*M-IL-6*	F: GTATGAACAACGATGATGCACTTG R: CTCTCTGAAGGACTCTGGCTTTG
*H-TNF-α*	F: CTGTAGCCCATGTTGTAGCAAAC R: TTGAAGAGGACCTGGGAGTAGAT
*M-TNF-α*	F: GACCCTCACACTCAGATCATCTT R: CCTTGAAGAGAACCTGGGAGTAG
*H-BAX*	F: GCTTCAGGGTTTCATCCAGGATC R: ATCCTCTGCAGCTCCATGTTACT
*M-BAX*	F: ATCCTCTGCAGCTCCATGTTACT R: TCATCCTCTGCAGCTCCATATTG
*H-Bcl-2*	F: GGATTGTGGCCTTCTTTGAGTTC R: CTTCAGAGACAGCCAGGAGAAAT
*M-Bcl-2*	F: GGATTGTGGCCTTCTTTGAGTTC R: CTTCAGAGACAGCCAGGAGAAAT
*H-caspase-9*	F: TGTCCTACTCTACTTTCCCAGGT R: CCCTTTCACCGAAACAGCATTAG
*M-caspase-9*	F: CCCGTGGACATTGGTTCTGG R: GAGGAAGGGCAGAAGTTCACA
*H-caspase-3*	F: TGAGCCATGGTGAAGAAGGAATAA R: CCCGGGTAAGAATGTGCATAAAT
*M-caspase-3*	F: CAGCCAACCTCAGAGAGACATT R: TTTCAGTTCAACAGGCCCATTTG
*H-SIRT2*	F: TGCGGAACTTATTCTCCCAGA R: GGAGAGCGAAAGTCGGGGAT
*M-SIRT2*	F: GCAGAACATAGACACGCTGG R: CCCTGGGAGTTGCTTCTGAGA
*H-β-actin*	F: CTAGGCGGACTGTTACTGAGC R: ATGTTTGCTCCAACCAACTGC
*M-β-actin*	F: AAATCTGGCACCACACCTTCTAC R: CAGCCTGGATAGCAACGTACAT

## Data Availability

The data that support the findings of this study are available from the corresponding author upon reasonable request. The RNA-sequencing data (SUB15288835) can be accessed in Sequence Read Archive (SRA) database of NCBI.

## Abbreviations

The following abbreviations are used in this manuscript:

JAC	Jaceosidin
SIMD	Sepsis-induced myocardial dysfunction
H3K18la	Histone H3K18 lactylation
PAMPs	Pathogen-associated molecular patterns
LPS	Lipopolysaccharide
TCM	Traditional Chinese medicine
DEGs	Differentially expressed genes
ECAR	Extracellular acidification rate
IF	Immunofluorescence
CHIP-qPCR	Chromatin immunoprecipitatin coupled with qPCR
LVEF	Left Ventricular Eiection Fraction
LVFS	Left Ventricular Fractional Shortening
LVDd	Left Ventricular end-diastolic internal diameter
LVDs	Left ventricular end-systolic internal diameters
H&E	Hematoxylin and eosin staining
TUNEL	TdT-mediated dUTP nick end labeling staining
cTnT	Cardiac troponin T
CK-MB	Creatine kinase isoenzymes
Kla	Lysine Lactylation
H3	Histone H3
H4	Histone H4
TNF-α	Tumour necrosis factor alpha
IL-6	Interleukin-6
IL-1β	Interleukin-1β
qPCR	Quantitative Real-time polymerase chain reaction

## References

[1] Rudd K.E., Johnson S.C., Agesa K.M., Shackelford K.A., Tsoi D., Kievlan D.R., Colombara D.V., Ikuta K.S., Kissoon N., Finfer S. (2020). Global, regional, and national sepsis incidence and mortality, 1990-2017: Analysis for the Global Burden of Disease Study. Lancet.

[2] Beesley S.J., Weber G., Sarge T., Nikravan S., Grissom C.K., Lanspa M.J., Shahul S., Brown S.M. (2018). Septic Cardiomyopathy. Crit. Care Med..

[3] Winterbottom F. (2022). Treating Sepsis in Patients with Heart Failure. Crit. Care Nurs. Clin. N. Am..

[4] Hollenberg S.M., Singer M. (2021). Pathophysiology of sepsis-induced cardiomyopathy. Nat. Rev. Cardiol..

[5] Lin Y., Xu Y., Zhang Z. (2020). Sepsis-Induced Myocardial Dysfunction (SIMD): The Pathophysiological Mechanisms and Therapeutic Strategies Targeting Mitochondria. Inflammation.

[6] Zindel J., Kubes P. (2020). DAMPs, PAMPs, and LAMPs in Immunity and Sterile Inflammation. Annu. Rev. Pathol..

[7] Maldonado R.F., Sa-Correia I., Valvano M.A. (2016). Lipopolysaccharide modification in Gram-negative bacteria during chronic infection. FEMS Microbiol. Rev..

[8] Li N., Zhou H., Wu H., Wu Q., Duan M., Deng W., Tang Q. (2019). STING-IRF3 contributes to lipopolysaccharide-induced cardiac dysfunction, inflammation, apoptosis and pyroptosis by activating NLRP3. Redox Biol..

[9] Cicchinelli S., Pignataro G., Gemma S., Piccioni A., Picozzi D., Ojetti V., Franceschi F., Candelli M. (2024). PAMPs and DAMPs in Sepsis: A Review of Their Molecular Features and Potential Clinical Implications. Int. J. Mol. Sci..

[10] Kuzmich N.N., Sivak K.V., Chubarev V.N., Porozov Y.B., Savateeva-Lyubimova T.N., Peri F. (2017). TLR4 Signaling Pathway Modulators as Potential Therapeutics in Inflammation and Sepsis. Vaccines.

[11] Tang J., Tam E., Song E., Xu A., Sweeney G. (2024). Crosstalk between myocardial autophagy and sterile inflammation in the development of heart failure. Autophagy Rep..

[12] Prescott H.C., Angus D.C. (2018). Enhancing Recovery From Sepsis: A Review. JAMA.

[13] Huang Y., Wang C., Zhou T., Xie F., Liu Z., Xu H., Liu M., Wang S., Li L., Chi Q. (2024). Lumican promotes calcific aortic valve disease through H3 histone lactylation. Eur. Heart J..

[14] Wang C., Xia Y., Qu L., Liu Y., Liu X., Xu K. (2021). Cardamonin inhibits osteogenic differentiation of human valve interstitial cells and ameliorates aortic valve calcification via interfering in the NF-kappaB/NLRP3 inflammasome pathway. Food Funct..

[15] Liu M., Li F., Huang Y., Zhou T., Chen S., Li G., Shi J., Dong N., Xu K. (2020). Caffeic Acid Phenethyl Ester Ameliorates Calcification by Inhibiting Activation of the AKT/NF-kappaB/NLRP3 Inflammasome Pathway in Human Aortic Valve Interstitial Cells. Front. Pharmacol..

[16] Zhang L., Zhang F., Li G. (2021). Traditional Chinese medicine and lung cancer—From theory to practice. Biomed. Pharmacother..

[17] Liu Y., Zhou L., Du B., Liu Y., Xing J., Guo S., Li L., Chen H. (2021). Protection against Doxorubicin-Related Cardiotoxicity by Jaceosidin Involves the Sirt1 Signaling Pathway. Oxid. Med. Cell. Longev..

[18] Gauthier T., Yao C., Dowdy T., Jin W., Lim Y.J., Patino L.C., Liu N., Ohlemacher S.I., Bynum A., Kazmi R. (2023). TGF-beta uncouples glycolysis and inflammation in macrophages and controls survival during sepsis. Sci. Signal.

[19] Sun Z., Song Y., Li J., Li Y., Yu Y., Wang X. (2023). Potential biomarker for diagnosis and therapy of sepsis: Lactylation. Immun. Inflamm. Dis..

[20] Wang C., Wang S., Wang Z., Han J., Jiang N., Qu L., Xu K. (2024). Andrographolide regulates H3 histone lactylation by interfering with p300 to alleviate aortic valve calcification. Br. J. Pharmacol..

[21] Wu J., Wang S., Gao P., Wang S., Yu H., Du Q., Liu M., Hou S., Jiang S., Xu H. (2025). Jatrorrhizine Alleviates Calcific Aortic Valve Disease via Interfering With Glycolysis Targeting ALDOA K42 Lactylation. Phytother. Res..

[22] Huang X.L., Wei X.C., Guo L.Q., Zhao L., Chen X.H., Cui Y.D., Yuan J., Chen D.F., Zhang J. (2019). The therapeutic effects of Jaceosidin on lipopolysaccharide-induced acute lung injury in mice. J. Pharmacol. Sci..

[23] Lee C.C., Yang C.Y., Su B.A., Hsieh C.C., Hong M.Y., Lee C.H., Ko W.C. (2020). The Hypotension Period after Initiation of Appropriate Antimicrobial Administration Is Crucial for Survival of Bacteremia Patients Initially Experiencing Severe Sepsis and Septic Shock. J. Clin. Med..

[24] Lee H., Jang D., Jeon J., Cho C., Choi S., Han S.J., Oh E., Nam J., Park C.H., Shin Y.S. (2020). Seomae mugwort and jaceosidin attenuate osteoarthritic cartilage damage by blocking IkappaB degradation in mice. J. Cell. Mol. Med..

[25] Ouyang Z., Li W., Meng Q., Zhang Q., Wang X., Elgehama A., Wu X., Shen Y., Sun Y., Wu X. (2017). A natural compound jaceosidin ameliorates endoplasmic reticulum stress and insulin resistance via upregulation of SERCA2b. Biomed. Pharmacother..

[26] Lee T.H., Jung H., Park K.H., Bang M.H., Baek N.I., Kim J. (2014). Jaceosidin, a natural flavone, promotes angiogenesis via activation of VEGFR2/FAK/PI3K/AKT/NF-kappaB signaling pathways in endothelial cells. Exp. Biol. Med..

[27] Mager C.E., Mormol J.M., Shelton E.D., Murphy P.R., Bowman B.A., Barley T.J., Wang X., Linn S.C., Liu K., Nelin L.D. (2023). p38 MAPK and MKP-1 control the glycolytic program via the bifunctional glycolysis regulator PFKFB3 during sepsis. J. Biol. Chem..

[28] Chen Y., Hu H., Wang C., Wu J., Zan J., Liu Y. (2025). Epigenetic Modulation by Lactylation in Sepsis: Linking Metabolism to Immune Dysfunction. J. Inflamm. Res..

[29] Wu D., Spencer C.B., Ortoga L., Zhang H., Miao C. (2024). Histone lactylation-regulated METTL3 promotes ferroptosis via m6A-modification on ACSL4 in sepsis-associated lung injury. Redox Biol..

[30] Li F., Si W., Xia L., Yin D., Wei T., Tao M., Cui X., Yang J., Hong T., Wei R. (2024). Positive feedback regulation between glycolysis and histone lactylation drives oncogenesis in pancreatic ductal adenocarcinoma. Mol. Cancer.

[31] Wang Y., Yang J., Hong T., Chen X., Cui L. (2019). SIRT2: Controversy and multiple roles in disease and physiology. Ageing Res. Rev..

[32] Sun L., Zhang Y., Yang B., Sun S., Zhang P., Luo Z., Feng T., Cui Z., Zhu T., Li Y. (2023). Lactylation of METTL16 promotes cuproptosis via m(6)A-modification on FDX1 mRNA in gastric cancer. Nat. Commun..

[33] Zhu W., Guo S., Sun J., Zhao Y., Liu C. (2024). Lactate and lactylation in cardiovascular diseases: Current progress and future perspectives. Metabolism.

[34] Qu L., Wang C., Xu H., Li L., Liu Y., Wan Q., Xu K. (2023). Atractylodin targets GLA to regulate D-mannose metabolism to inhibit osteogenic differentiation of human valve interstitial cells and ameliorate aortic valve calcification. Phytother. Res..

[35] Zhang D., Tang Z., Huang H., Zhou G., Cui C., Weng Y., Liu W., Kim S., Lee S., Perez-Neut M. (2019). Metabolic regulation of gene expression by histone lactylation. Nature.

[36] Yu H., Du Q., Wu J., Feng F., Hou S., Liu M., Wang S., Liu X., Wang C., Xu K. (2025). Gastrodin regulates H3K14la through the CDT2-KAT2A axis to treat Sepsis-induced myocardial dysfunction. Int. Immunopharmacol..

[37] Zhang M., Zhu C.X., Luo Z.Y., Liu J.H., Khan M.T., Sun Y.W., Wei D.Q., Zhang Y.J. (2023). Exploring Key Proteins, Pathways and Oxygen Usage Bias of Proteins and Metabolites in Melanoma. J. Comput. Biophys. Chem..

[38] Ndongson-Dongmo B., Lang G.P., Mece O., Hechaichi N., Lajqi T., Hoyer D., Brodhun M., Heller R., Wetzker R., Franz M. (2019). Reduced ambient temperature exacerbates SIRS-induced cardiac autonomic dysregulation and myocardial dysfunction in mice. Basic Res. Cardiol..

[39] Yao Y.Y., Bian L.G., Yang P., Sui Y., Li R., Chen Y.L., Sun L., Ai Q.L., Zhong L.M., Lu D. (2019). Gastrodin attenuates proliferation and inflammatory responses in activated microglia through Wnt/beta-catenin signaling pathway. Brain Res..

[40] Wang W., Wang Y., Wang F., Xie G., Liu S., Li Z., Wang P., Liu J., Lin L. (2024). Gastrodin regulates the TLR4/TRAF6/NF-kappaB pathway to reduce neuroinflammation and microglial activation in an AD model. Phytomedicine.

